# Fat Wasting Is Damaging: Role of Adipose Tissue in Cancer-Associated Cachexia

**DOI:** 10.3389/fcell.2020.00033

**Published:** 2020-02-12

**Authors:** Xiaoting Sun, Xiaogang Feng, Xiaojing Wu, Yongtian Lu, Kaihong Chen, Ying Ye

**Affiliations:** ^1^Department of Medical Oncology, Shuguang Hospital, Shanghai University of Traditional Chinese Medicine, Shanghai, China; ^2^Institute of Physiology, University of Zurich, Zurich, Switzerland; ^3^Department of Cardiology, Shenzhen University General Hospital, Shenzhen, China; ^4^Department of ENT, Shenzhen Second People’s Hospital, The First Affiliated Hospital of Shenzhen University, Shenzhen, China; ^5^Department of Cardiology, The Affiliated Longyan First Hospital of Fujian Medical University, Longyan, China; ^6^Department of Oral Implantology, School and Hospital of Stomatology, Tongji University, Shanghai Engineering Research Center of Tooth Restoration and Regeneration, Shanghai, China

**Keywords:** adipose tissue, browning, lipolysis, thermogenesis, cancer cachexia

## Abstract

Loss of body weight, especially loss of adipose tissue and skeletal muscle weight, characterizes cancer-associated cachexia (CAC). Clinically, therapeutic options for CAC are limited due to the complicated signaling between cancer and other organs. Recent research advances show that adipose tissues play a critical role during thermogenesis, glucose homeostasis, insulin sensitivity, and lipid metabolism. Understanding the adipocyte lipolysis, the formation of beige adipocytes, and the activation of brown adipocytes is vital for novel therapies for metabolic syndromes like CAC. The system-level crosstalk between adipose tissue and other organs involves adipocyte lipolysis, white adipose tissue browning, and secreted factors and metabolites. Novel CAC animal models and accumulating molecular signaling knowledge have provided mechanisms that may ultimately be translated into future therapeutic possibilities that benefit CAC patients. This mini review discusses the role of adipose tissue in CAC development, mechanism, and therapy.

## Introduction

Cancer-associated cachexia (CAC) has a unique tumor-driven pattern that can lead to progressive functional impairment, treatment-related complications, poor quality of life, and mortality. It is defined by an ongoing loss of skeletal muscle mass, with or without loss of fat mass, that can be partially but not entirely reversed by conventional nutritional support (Fearon et al., 2011). The diagnosis of CAC is based on the speed of weight loss and the BMI index. Recent assessments have suggested that CAC affects 60–80% of advanced cancer patients and directly causes at least 20% of cancer death (Stewart et al., 2006). CAC patients are more susceptible to the toxic effects of anti-tumor therapies such as chemotherapy, while the increased toxicity requires drug withdrawal and dose reduction in cancer treatment, and it therefore increases morbidity and mortality. This fatal disease can be divided into three typical stages: pre-cachexia, cachexia, and refractory cachexia (Argiles et al., 2011). Patients with CAC have significant weight loss, anorexia, anemia, fatigue, intestinal malabsorption, nausea, profound endocrine alterations, and metabolic chaos, with severe disruption of protein, lipid, and carbohydrate metabolism (Tisdale, 2002).

It is agreed that CAC is an energy balance disorder in which the tumor competes with other organs and tissues for fuel and biosynthetic substrates (Argiles et al., 2014). The intrinsic metabolic rate defined by aerobic versus anaerobic energy metabolism is essential for CAC progression. Various energy expenditure-related pathways are suggested in the development of CAC. About 40 years ago, researchers found that futile cycles that reduce efficiency in adenosine triphosphate (ATP) usage contribute to the hypermetabolism. For example, the Cori cycle, a lactate–glucose carbon recycling system between the muscle and liver, may nourish the tumor (Holroyde and Reichard, 1981). Recently, leakiness of the respiratory electron-transport chain is found to confer high energy expenditure. Instead of ATP synthase, Uncoupling protein 1 (UCP1), which is only expressed in brown or beige adipocytes, speeds up respiration and converts electrochemical energy into heat production (Bing et al., 2000). Moreover, other mitochondria abnormities, such as atrophy of oxidative capacity (Julienne et al., 2012), may be involved in muscle weight loss.

As a multi-organ syndrome, CAC is closely associated with skeletal muscle, adipose tissue, the bone, the liver, the neural system, and the gut (Argiles et al., 2018). Among these organs, the communication between the adipose tissue and tumor is under intense investigation. Although studies have suspected the relationship between the adipose tissue and tumor for over 30 years (Brooks et al., 1981; Shellock et al., 1986), the detailed mechanisms have only recently been discovered. Adipose tissue is one of the largest organs in humans. Besides energy storage, accumulating evidence demonstrates its unveiled roles within endocrinology, energy consumption, thermogenesis, stem cell pool, neuronal differentiation, and inflammatory regulation. However, its crosstalk with the tumor is largely overlooked. In humans, white adipose tissue (WAT) is mainly composed of large spherical adipocytes, in which a unilocular lipid droplet occupies most of the cell volume. WAT possesses endocrine and paracrine functions and stores energy in the form of triglycerides. In comparison, brown adipocytes in brown adipose tissue (BAT) exhibit a substantial amount of mitochondria and scattered cytoplasmic droplets. The most distinguished signature in BAT is its high-level expression of mitochondrial UCP1, which is the key to lipid oxidation and thermogenesis. Another type of adipocyte, the “beige” adipocyte, originated from white adipocyte but expresses UCP1, shows plasticity, and can be transited from white adipose tissue by various signals (Rosen and Spiegelman, 2006). Clinically, WAT browning and BAT activation are considered as promising methods for combating obesity and metabolic syndromes. On the contrary, similar activities under local or distant stimuli in cancer make them potential causes of CAC. In this review, we focus on the role of adipose tissue dysfunction in CAC and review the molecular mechanism that underlies it. An adequate understanding of this system-level adipose tissue crosstalk will be beneficial for novel treatments for CAC.

### WAT Lipolysis in CAC

The white adipocyte is the major storage space for triacylglycerol (TAG), and the balance of lipolysis/lipogenesis maintains the dynamic homeostasis in the adipocyte, as well as guides the systemic energy production in CAC. Independent of malnutrition, adipocyte lipolysis is strongly involved in CAC, inducing lipid loss (Ryden et al., 2008). Lipolytic factors or hormones, such as tumor necrotic factor α (TNFα) (Oliff et al., 1987), interleukin-6 (IL-6) (Strassmann et al., 1992), Zinc-α2-glycoprotein (ZAG) (Bing et al., 2004), catecholamines, and natriuretic peptides (Kalra and Tigas, 2002), explain lipolysis in cancer cachexia. Following this line, recent studies show that two key lipases (Agustsson et al., 2007; Das et al., 2011), which break down the fat, mediate CAC, and serve as potential targets for CAC treatment. Other than the canonical pathways, lipolysis can also be regulated by degradation of perilipin, a lipid droplet-associated packaging protein (Kovsan et al., 2007). However, its relationship with CAC warrants further investigation. Besides, *de novo* lipogenesis is reduced in tumor-bearing animals (Trew and Begg, 1959). Lipogenic enzymes, such as lipoprotein lipase (LPL) and fatty acid synthase (FAS), are significantly reduced in the adipose tissue adjacent to the tumor (Notarnicola et al., 2012), validating the tumor-supporting role of WAT in CAC. Other than lipolysis *per se*, inflammation is a well-known driving force for WAT lipolysis. CAC is correlated with profound inflammation, which may, in turn, stimulates WAT loss, suggesting inflammatory cytokines may serve as biomarkers for CAC diagnosis. Indeed, animal models show a strong correlation between tumor presence and elevated serum inflammatory cytokines (Das et al., 2011). However, the association between serum cytokines and fat loss in patients is somewhat ambiguous (Blum et al., 2011), probably due to the transient and diverse-origin nature of serum cytokines. Though further validation is needed, lipolysis-related CAC biomarkers in peripheral blood are particularly important for diagnosis.

Reserving excess lipids and regulating circulating fatty acid (FA) to other organs are major functions of the white adipocyte. During CAC, lipolysis activation in WAT may increase the circulating FA. Consequently, lipid overload may process a secondary effect on various organs. It is suspected that tumor benefits from the releasing FA during CAC. Indeed, in an acute fasting model, circulating FA greatly increased tumor proliferation (Sauer et al., 1986). In accordance with this, our work showed that a hypoxia-induced metabolic shift promotes tumor FA importation and β-oxidation (Iwamoto et al., 2018). In other non-adipose organs, lipotoxicity is also widely reported in liver, skeleton muscle, pancreas, and heart (van Herpen and Schrauwen-Hinderling, 2008).

### Browning in CAC

In WAT, sympathetic stimulations, such as cold exposure or a β3 agonist, strongly increase thermogenic beige adipocytes. This process is termed as “browning”. Various recent studies have reported that an upregulated browning process promotes energy expenditure and CAC (Kir et al., 2014; Petruzzelli et al., 2014). By neutralizing the browning stimulator parathyroid hormone-related protein (PTHrP), CAC is ameliorated and fat loss is rescued in animal models (Kir et al., 2014). Additionally, jeopardizing the tumor-derived exosome, which is a vehicle for possible browning stimulators, rescued fat loss in tumor-bearing mice (Hu et al., 2018). These findings suggested the anti-CAC effect of the browning blockade. Understanding the mechanism of beige cell differentiation and UCP1 production is crucial for combating CAC. During the last decade, the transcriptional regulation of UCP1 has been elucidated. Peroxisome proliferator-activated receptor γ (PPARγ) (Rosen et al., 2002), PPARγ coactivator 1α (PGC-1α) (Puigserver et al., 1998), PR domain containing 16 (PRDM16) (Seale et al., 2007), and other transcription factors are responsible for UCP1 production in brown and beige adipocytes. Other than that, extracellular stimulations, such as catecholamines (Nguyen et al., 2011), crotamine (Marinovic et al., 2018), prostaglandins (Vegiopoulos et al., 2010), fibroblast growth factor 21 (FGF21) (Dutchak et al., 2012), ZAG (Elattar et al., 2018), Bone morphogenetic proteins (BMPs) (Tseng et al., 2008), and alcohol-retinoic acid axis (Wang et al., 2017), have recently been reported to elevate the browning process and facilitate CAC. During the browning process, beige cells *de novo* originated from a smooth muscle cell-like lineage and can be converted back to the “white-like” phenotype (Rosenwald et al., 2013; Wang et al., 2013), while PRDM16 (Long et al., 2014) and BMP7 (McDonald et al., 2015) serve as strong stimulators for their differentiation. Although various mechanisms, such as autophagy, microflora, exosome, and long non-coding RNAs, have been reported to be involved in the WAT browning process, whether or not this confers CAC is not fully validated. Only a handful of studies exhibited an anti-cachexia effect in limited animal models. Future studies should focus on these targets from a clinical point of view.

Interestingly, white adipose depots show heterogeneity in browning efficiency. Certain depots, such as inguinal WAT, are sensitive to browning stimulation, while visceral fat depots are resistant to browning. The latter was previously identified as being “true white adipose tissue” and harmful. Considering the browning ability differences, do adipose depots contribute to CAC differently? It has been reported that visceral fat may switch its phenotype for browning under certain stimulations (Yang et al., 2017), though the switching mechanism is still not well understood. This interesting question warrants further investigation and may improve our understanding of the mechanism of browning-conferred CAC.

### BAT Activation in CAC

BAT depots are highly vascularized, and the interscapular site is the main location for BAT in rodents (Rosen and Spiegelman, 2014). Except for specific markers, such as the Zinc finger in the cerebellum 1 (Zic1), brown adipocytes share overlapping gene signatures with beige adipocytes (Walden et al., 2012). Compared with beige cells, brown adipocytes have a higher basal level of UCP1 expression (Wu et al., 2012). From a developmental point of view, brown adipocytes are marked by transcription factors myogenic factor 5 (Myf5) (Seale et al., 2008) and paired box 7 (Pax7) (Lepper and Fan, 2010), similarly to myogenic precursor cells. Brown fat precursor cells that express early B cell factor 2 (EBF2) and platelet-derived growth factor receptor α (PDGFRα) process *de novo* differentiation into mature brown adipocytes (Wang et al., 2014). β1-adrenergic receptor (ADRB1) also mediates norepinephrine-induced *de novo* brown adipogenesis in BAT (Lee et al., 2015). Interestingly, ADRB1 expression is correlated with the lipolytic rate in CAC patients (Cao et al., 2010), suggesting that a BAT blockade may be a potential therapy for CAC. Although speculated for a long time (Shellock et al., 1986), the clinical evidence that BAT contributes to CAC is limited. This may be due to the small amount and sporadic distribution of adult BAT in humans as well as restrictions of current imaging methods to describe BAT and quantify its function.

Interestingly, loss of brown adipocytes may sequentially induce WAT browning, indicating a compensatory mechanism between mature brown and beige adipocyte (Schulz et al., 2013). It would be exciting to identify whether this mechanism exists in CAC.

### Adipocyte–Non-adipocyte Crosstalk in CAC

As a multi-functional organ, adipose tissue communicates with various cell types. In the context of CAC, the adipocyte-myocyte, adipocyte-cancer cell, and adipocyte-inflammatory cell crosstalk have received particular attention. Firstly, adipocytes and skeletal muscle communicate in CAC: (1) brown adipocyte share the same lineage origin with skeletal muscle and may respond to similar signals (Seale et al., 2008); (2) the fibro-adipogenic precursor in the muscle may differentiate into white adipocyte in CAC (Stephens et al., 2011); (3) adipocyte-derived cytokines stimulate muscle atrophy (Pellegrinelli et al., 2015); and (4) myokines, such as irisin and FGF21, promote browning and fat loss (Bostrom et al., 2012; Veniant et al., 2015). Secondly, adipose tissue is strongly associated with inflammatory cells. In CAC patients, systemic inflammation is one of the major driving forces for adipose wasting. Released by tumor cells and activated immune cells, inflammatory cytokines, such as ZAG (Elattar et al., 2018) and TNFα (Patel and Patel, 2017), promote adipose wasting in CAC. Moreover, direct immune cell–adipocyte interaction may also drive cachexia (Baazim et al., 2019). Interestingly, in bone-marrow, adipocytes negatively regulate surrounding myeloid cells (Naveiras et al., 2009). However, in an adipose-wasting setting, such as anorexia nervosa, bone-marrow adipocytes do not undergo lipolysis but paradoxically expand (Bredella et al., 2009). These interesting findings need to be investigated in CAC. Thirdly, as previously mentioned, adipocyte-tumor cell communication includes adipocyte lipolysis, which stimulates tumor growth (Sauer et al., 1986), and tumor cell-derived cytokines or hormones that drive the browning process (Kir et al., 2014; Petruzzelli et al., 2014; Elattar et al., 2018).

**FIGURE 1:**
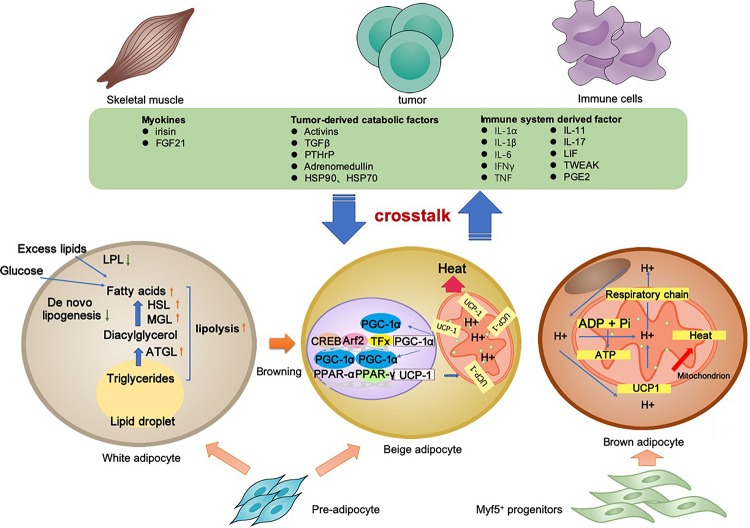
Adipocyte-associated crosstalk influences metabolic homeostasis in CAC. In CAC, WAT undergoes increased lipolysis and reduced lipogenesis, which results in adipose tissue loss. In addition to lipolysis, white adipocyte browning and brown adipocyte activation stimulate UCP1 upregulation for thermogenesis and high energy expenditure. The adipocyte–myocyte, adipocyte–cancer cell, and adipocyte–inflammatory cell crosstalk influences metabolic homeostasis. Tumor-derived factors, adipokines, myokines, and other factors are all involved in the lipolysis and/or browning in adipose tissues. The adipose tissue wasting also has an effect on other organs through various pathway. Collectively, these changes result in a negative energy balance, which contributes to the development and progression of CAC.

Cancer-associated cachexia is a syndrome involving multiple organs and tissues. Between the adipocyte and various non-adipocytes, direct and indirect communication exists in the context of CAC. The molecular mechanisms of lipolysis and thermogenesis in three types of adipocytes, as well as the adipose tissue crosstalk, are summarized in Figure 1.

### Animal Models Used in CAC Research

Appropriate animal models are crucial for understanding the mechanisms for clinical therapy. Over the years, various animal models have been proposed to mimic CAC. Due to the complexity of CAC, animal models can be characterized by loss of muscle, loss of adipose tissue, systemic inflammation, or anorexia. During the last decade, tumor-bearing rodent models have been upgraded. Genetically modified animal models have been developed for better mimicking the clinical situation. New animal species, such as zebrafish, are generated for CAC studies. Here, we review the commonly used animal model for CAC in Table 1, with detailed information on animal species, genetic modification, experimental period, weight loss, muscle loss, and, importantly, adipose tissue wasting.

**TABLE 1:** Animal models used in cancer-associated cachexia research.

**Animal model**	**Host**	**Experimental period (Day)**	**Weight loss (%)**	**Skeletal muscle wasting**	**Adipose tissue wasting**	**References**
Lewis lung carcinoma (LLC)	C57BL/6 mice	15	6.6	Yes (with reduced cross-sectional areas of muscle fibers)	Yes (with UCP1 increased in BAT)	Puppa et al., 2014; Llovera et al., 1998
C26 colorectal carcinoma	BALB/c mice; CD2F1 mice	20	18	Yes (20–30% with reduced cross-sectional areas of muscle fibers)	Yes (70% reduction with UCP1 increased in BAT)	Bonetto et al., 2016; Murphy et al., 2012
Yoshida hepatoma (AH-130)	Wistar rats	15	35	Yes (protein loss in gastrocnemius and heart muscle)	Yes	Hoshino, 1963; Tessitore et al., 1993; Tschirner et al., 2012
Walker 256 mammary adenocarcinoma	Sprague-Dawley rats; Wistar rats	14–21	6	Yes (with gastrocnemius muscle reduced)	Yes (with decreased eWAT)	Guaitani et al., 1982
Murine adenocarcinoma 16 (MAC16)	NMRI mice	18–30	20–33	Yes (20% with UCP-2 and -3 increased in skeletal muscle)	Yes (67% reduction with UCP1 increased in BAT)	Bibby et al., 1987; Bing et al., 2000
Apc^min/+^	C57BL/6 mice	84–140	20–25	Yes (with reduced mitochondrial content in gastrocnemius muscle)	Yes (with decreased eWAT)	Baltgalvis et al., 1985; Puppa et al., 2011
Tsc2^+/–^Eμ-Myc lymphoma	C57BL/6 mice	9–20	∼20	Yes (significant loss of muscle mass)	Yes (complete loss of adipose tissue)	Robert et al., 2012
MKN45c185 and 85As2 Stomach cancer	F344/NJcl-rnu/rnu rats	28	4–8	Yes	Yes	Takeda et al., 2008; Fujitsuka et al., 2011; Yanagihara et al., 2013; Terawaki et al., 2014
MDA-MB-231 breast cancer bone metastasis	Nude mice	20	12	Yes (with gastrocnemius, tibialis anterior, and extensor digitorum longus muscle decreased)	/	Hain et al., 2019
ASV-B hepatocellular carcinoma	C57BL/6 mice	119	34	Yes (decreased in mass of gastrocnemius, tibialis anterior, and extensor digitorum longus muscle)	Yes (with decreased eWAT)	Erdem et al., 2019
S2-013, PANC1, Pa04 pancreatic cancer	nude mice	10	6–15	Yes (with reduced cross-sectional areas of muscle fibers)	Yes	Shukla et al., 2014, 2015; Winnard et al., 2016
KRAS^G12D/+^ P53^R172H/+^ Pdx-Cre (KPC) mouse	C57BL/6 mice	5–14	/	Yes (4.5–7.7% reduction)	Yes (52.6–69% reduction. With UCP1 decreased in WAT and BAT)	Michaelis et al., 2017
Kras^+/G12D^ Ptf1a^+/ER–Cre^ Pten^f/f^ (KPP) mouse	C57BL/6 mice	107	∼25	Yes (decreased tibialis anterior, quadriceps femoris, and gastrocnemius muscle masses)	Yes (with eWAT and iBAT decreased)	Talbert et al., 2019
Kras^G12V^-induced hepatocellular carcinoma	Zebrafish	28	30	Yes (increased level of fibrosis along with the loss of muscle fibers)	/	Yang et al., 2019
Scrib Ras^V12^ tumor	Drosophila	5	/	Yes (muscle ATP levels reduced)	Yes (fat body marked reduction)	Figueroa-Clarevega and Bilder, 2015

Due to the heterogeneity of human cancers, none of the experimental models are suitable for the complete recapitulation of the clinical CAC features. Xenograft mouse models are easy to establish. However, fast tumor progression normally masks the CAC progression. Genetic models recapitulate the oncogenesis process and some genetic features in the clinic, while the non-stable occurrence rate limits further studies. In general, experimental mammal CAC models are useful for investigating adipose tissue–tumor communications, especially BAT-tumor communications. While the zebrafish or drosophila models may not perfectly reflect CAC features, their fast generating time and the convenience with which to produce the wild type or mutants make them attractive for drug screens. Notably, there is a lack of guidelines and consensus in CAC models, even in the most common models. Due to the non-standardized experimental conditions and CAC outcome measurements, the same model may behave differently. It increases the complexity of this field (Penna et al., 2016).

### Therapeutic Advances

Medical interventions for cachexia are limited and urgently warranted. Nutritional supports involve caloric intake, marine n-3 fatty acids, amino acid, and micronutrients. The evidence of this nutrition supports is limited. Apart from this, clinically, there has been a lack of a standard for nutrition support until now (Gullett et al., 2011).

Anti-CAC drug therapy has been continuously proposed over the last three decades. Appetite stimulants, such as megestrol acetate or tetrahydrocannabinol, promote food intake but show no effect on survival (Jatoi et al., 2002; Lesniak et al., 2008). Anabolic factors aiming to maintain body mass have been proposed for CAC. Hormones such as oxandrolone increase lean body mass but not fat body mass in CAC patients (Lesser et al., 2008). Ghrelin, a growth hormone stimulator secreted by the stomach, shows significant improvement in lean body mass (Garcia et al., 2007). A muscle-specific androgen receptor modulator improves lean body mass but failed in later larger trials (Dalton et al., 2011). Anti-inflammatory drugs, such as NSAIDs, improve weight gain (Lai et al., 2008). However, the TNFα inhibitor does not show clinical significance in CAC patients (Jatoi et al., 2010). Recently, various targets, including muscle stem cells (Rinaldi and Perlingeiro, 2014), statins (Palus et al., 2013), mitochondria (van der Ende et al., 2018), and microbiota (Fukawa et al., 2016), have been proposed for novel therapeutic options. However, clinically, only a handful of drugs are approved for treating CAC patients. The majority of them are appetite stimulants. Notably, Anamorelin, a promising selective agonist of ghrelin, significantly increases lean body mass, but did not affect muscle strength or quality of life in a phase III trial (Temel et al., 2016), and it was thus rejected by the European Medicines Agency in 2017.

Targeting the adipose tissue for CAC therapy has been proposed but not yet proven in the clinic. New therapies that block adipose tissue lipolysis and/or thermogenesis can potentially be used to treat cachexia.

## Conclusion

Cancer-associated cachexia is a chronic disease with multiple organs or tissues involved. Clinically, a novel therapy is warranted for not only achieving weight gain but also increasing the quality of life. Recent advances highlight the adipose tissue involvement in CAC. Among adipose tissues, WAT guides systemic energy production via the balance between lipogenesis and lipolysis. BAT has recently been identified in adult humans with profound physiopathological functions. The browning process stimulates beige adipocyte differentiation and thermogenesis. These adipose tissues contribute to CAC differently. During CAC development, adipose tissues crosstalk with other cell types or organs and exhibit therapeutic potential. In this field, the key issues remain: (1) How are white/beige/brown adipocytes regulated in CAC pathological status? (2) What are the molecular details for adipocyte-non-adipocyte communications in CAC? (3) Are there better animal models for investigating adipose tissues in CAC? (4) What is the system-level landscape for adipose tissues in CAC? Altogether, the ultimate goal in this field will be to identify new targets in adipose tissues for treating CAC. The mechanistic studies need to be validated in clinical trials and translated into therapies for combating CAC.

## Author Contributions

XS and YY generated the ideas, reviewed publications, and wrote the manuscript. XF, XW, YL, KC, and YY participated in discussions.

## Conflict of Interest

The authors declare that the research was conducted in the absence of any commercial or financial relationships that could be construed as a potential conflict of interest.
